# One or two pups - optimal reproduction strategies of common noctule females

**DOI:** 10.1186/s40850-022-00119-8

**Published:** 2022-04-02

**Authors:** Katerina Zukalova, Veronika Seidlova, Vladimir Piacek, Monika Nemcova, Michal Pribyl, Jiri Pikula, Jan Zukal

**Affiliations:** 1 Department of Ecology and Diseases of Zoo Animals, Game, Fish and Bees, University of Veterinary Sciences Brno, Palackého tř. 1946/1, 612 42 Brno, Czech Republic; 2 Institute of Vertebrate Biology of the Czech Academy of Sciences, Květná 8, 603 65 Brno, Czech Republic; 3 Department of Botany and Zoology, Masaryk University, Kotlářská 267/2, 611 37 Brno, Czech Republic

**Keywords:** Gestation, Progesterone, Body weight, Embryo resorption, Chiroptera

## Abstract

**Background:**

The success of animal reproduction is impacted by a trade-off between energetic costs and mortality associated with immediate vs. future reproductive attempts. The reproductive strategies of European insectivorous bats differ from common mammalian standards due to the use of delayed fertilisation. Phenology of bat reproduction, including length of pregnancy, which may vary in the same species at different latitudes, between years at the same site or between individuals within a colony, is influenced by ecological conditions. To assess factors influencing the course of pregnancy, we evaluated levels of blood progesterone in 20 female common noctule bats *Nyctalus noctula*. The bats were individually tagged and randomly divided into two groups with different hibernation ending points (i.e. a control group vs. a treatment group with one-week longer hibernation). Following emergence from hibernation, the bats were kept in a wooden box at a stable temperature of 22 °C.

**Results:**

The majority of females gave birth to a single neonate (65%), but one female aborted her pups 2 days before the first successful births of other females. Based on development of progesterone concentration, we were able to define a number of different reproduction strategies, i.e. females with single offspring or twins, and females with supposed resorption of one embryo (embryonic mortality after implantation of the developing fertilised egg). Progesterone levels were much higher in females with two embryos during the first part of gestation and after birth. Progesterone levels were at their highest mid-gestation, with no difference between females carrying one or two foetuses. Length of gestation differed significantly between the two groups, with the longer hibernation (treatment) group having a roughly two-day shorter gestation period.

**Conclusions:**

Female *N. noctula* are able to manipulate their litter size to balance immediate and future reproduction success. The estimated gestation length of approx. 49-days appears to be standard for *N. noctula*, with females optimising their thermoregulatory behaviour to keep the length of gestation as close to the standard as possible.

**Supplementary Information:**

The online version contains supplementary material available at 10.1186/s40850-022-00119-8.

## Background

Life-history characteristics among mammals tend to vary with body size, with small mammals generally having large litters of small neonates that grow and mature rapidly but are short-lived, and large mammals having small litters and young that grow and mature slowly but have a long reproductive lifespan [1]. Bats are unusual in that they are small mammals with a slow life-history and most species produce one litter each year of one or two young which develop slowly over a relatively long gestation [2]. In the temperate zone, proper timing of reproduction plays a very important role. A key concept of life-history theory is the trade-off between energetic costs and mortality risk resulting from immediate reproduction vs. the potential success of future reproductive attempts [3]. A mammal female may terminate a reproductive attempt that has a low probability of success and threatens future reproduction by blocking implantation, resorbing embryos, aborting foetuses or ceasing lactation [4].

The common noctule bat *Nyctalus noctula* is a common and widely distributed Palaearctic species with maternity colonies located mainly in the northern geographic areas of its range. These large, fast-flying insectivorous bats are resident in the Czech Republic throughout the year. Its reproduction is subject to the rules of delayed ovulation by hibernating temperate bat species sensu [5], whereby viable spermatozoa are stored in uterine horns during hibernation. Ovulation occurs after arousal from late March to mid-April, depending on the current weather conditions [6, 7]. It is generally accepted that gestation length is not fixed in bats due to the prevalence of torpor length variation. Time of parturition may also vary between individuals of the same species in a single season [8–10], depending on latitude [11–17], or between climatically different years [18–22]. When emerging from hibernation, the female is already in early gestation and it can be difficult to cover energetic costs during a fluctuating food supply [23]. To survive unpredictable environmental conditions or lack of food, bats may enter a period of torpor [24]. On the other hand, entering a period of torpor will delay certain physiological processes, including foetal development [25]. Females may then adopt thermoregulatory behaviours that optimise juvenile development [26]. Juvenile bats in temperate regions must learn to fly, produce and process echolocation calls, capture prey and store fat in preparation for hibernation; hence, it is advantageous for parturition to occur as early as possible in order to allow post-reproductive females and young the maximum time to prepare for winter [27]. In nature, *N. noctula* give birth to one or two neonates from late June to early July [7, 28], corresponding to a gestation period of ca. 73 days [29]. Similar gestation period was registered by Pikula et al. [28] in captivity under microclimatic conditions mimicking natural fluctuation of temperature. Under laboratory conditions, where food is not limited and ambient temperatures are favourable and stable, gestation periods may be closer to 49 days [11].

Gestation is maintained by the concentration of sex hormones, which reflect gonadal activity [30]. In vespertilionids, concentration of progesterone has only been reported in females of six species, i.e. the Mexican free-tailed bat *Tadarida brasiliensis* [31], the common pipistrelle *Pipistrellus pipistrellus* [25], the little brown bat *Myotis lucifugus* [32, 33], Gould’s wattled bat *Chalinolobus gouldii* [34], the Arafura large-footed bat * Myotis mollucarum* [35] and Schreiber’s long-fingered bat *Miniopterus schreibersii* [36]. Data acquisition for such studies is difficult as blood must be sampled invasively and the amount of material thus collected is small. Moreover, it is impossible to perform repeated sampling from the same individual over a short period of time due to the small body size of insectivorous bats [37].

Recently, we had an unparalleled opportunity to monitor pregnancy in female *N. noctula*, including progesterone levels, after rescuing into veterinary care a cluster of 20 bats from a building undergoing reconstruction. In order to better understand female bat reproductive physiology, we manipulated the point at which some of the bats ceased hibernating, providing two groups that differed in emergence by 7 days, allowing us to focus on factors influencing the course of pregnancy in individual females. We hypothesised that 1) females will optimize their reproduction strategy based on available energy resources here expressed as body mass after hibernation, and 2) that gestation period would be similar in both experimental groups due to a lack of variation in ecological impacts (e.g. weather conditions, food supply) during the foetal development.

## Results

### Progesterone pattern and determination of the three reproductive strategies

**Fig. 1:**
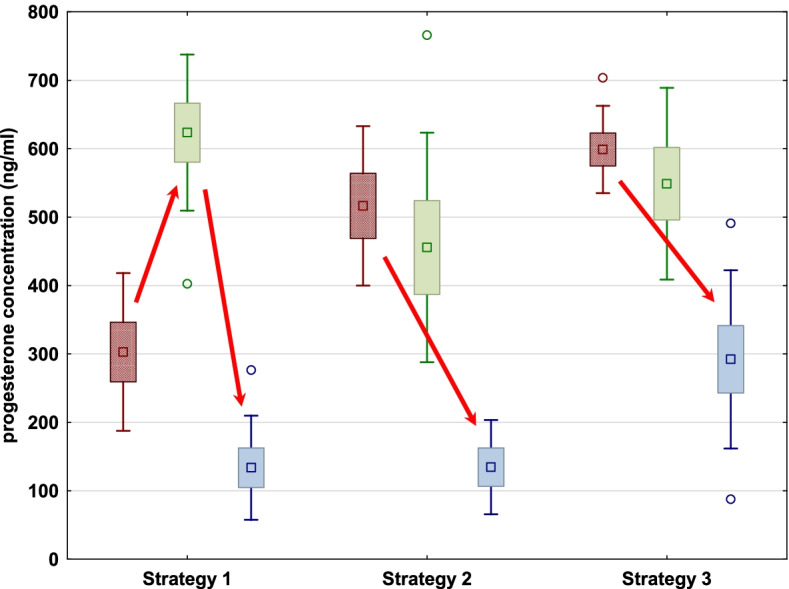
Changes in blood progesterone concentration in *Nyctalus noctula* females utilising three different reproductive strategies. Square = mean, box = standard error, whisker = standard deviation, dot = outliers. Red arrows demonstrate the trend in progesterone concentration change

The majority of females from both groups (*n* = 7, 65%) gave birth to a single neonate, while one female from the control group aborted her pups 2 days before the first successful births by other females. K-means clustering conclusively separated the cluster of seven females with single young and the standard progesterone pattern, i.e. low concentrations at the beginning of gestation and after birth, and highest concentrations in mid-gestation (Fig. 1). Two other clusters consisted of females with either single young or twins with a declining progesterone pattern. As such, three strategies could be defined on the basis of cluster analysis results: 1) females that gave birth to single young, 2) females that gave birth to single young but have a progesterone pattern typical of twins, and 3) females that gave birth to twins.

**Fig. 2:**
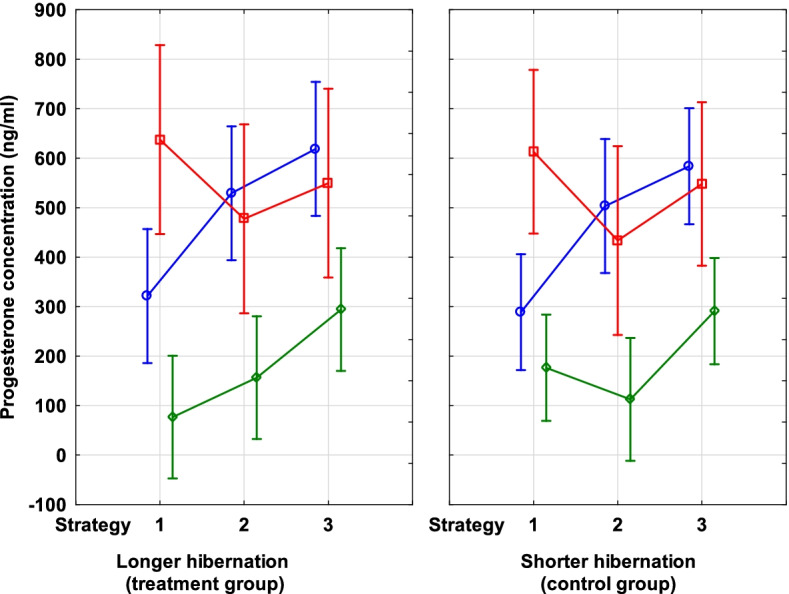
Results of Main Effects ANOVA. Vertical bars denote 95% confidence intervals. Blue = Stage 1 (initial stage of pregnancy), red = Stage 2 (late stage of pregnancy), green = Stage 3 (stage shortly after delivery)

Factorial ANOVA confirmed a difference in progesterone concentration between the three groups; however, the impact of treatment (i.e. length of hibernation) was not significant (Table 1). Progesterone concentration only did not differ significantly during mid-gestation (Stage 2), with post-hoc comparisons indicating that females giving birth to single young had the lowest progesterone concentration at Stage 1 and females giving birth to twins having highest progesterone concentrations after delivery (Stage 3; Fig. 2). Moreover, progesterone concentration was positively correlated with the post-hibernation body weight of females only at Stage 1 (*r* = 0.462, *p* = 0.040).

**Table 1:** Main Effects ANOVA confirmed significant differences in progesterone concentration between the three reproductive strategies (defined through k-means clustering), but found no influence of hibernation length

Effect	Value	F	Effect df	Error df	***p***
Intercept	0.026	174.89	3	14	**< 0.001**
Length of hibernation	0.964	0.17	3	14	0.914
Reproductive strategy	0.120	8.81	6	28	**< 0.001**

### Parameters determining the three reproduction strategies

While the three female strategies defined above were evenly distributed within the control (4, 3 and 4 females, respectively) and treatment (3, 3 and 3 females, respectively) groups, age structure was not balanced. Six subadult females were reproducing and all except one gave birth to single young (two with suspected embryo resorption). The one exception was bearing twins, which were aborted 2 days before the first normal births. As we found no size difference (forearm length or body weight) between adult and subadult females (Table 2), they were pooled for future analyses.

**Table 2:** T-tests found no difference in the size of adult and subadult females, measured as the length of antebrachium (LAt) and body weight

Variable	Mean adult	Mean subadult	t-value	df	p	Valid N adult	Valid N subadult	SD adult	SD subadult	F-ratio variances	p variances
LAt	54.62	54.08	0.97	18	0.343	14	6	1.08	1.26	1.36	0.601
Body weight	21.68	21.17	0.52	18	0.611	14	6	1.90	2.34	1.52	0.502

**Fig. 3:**
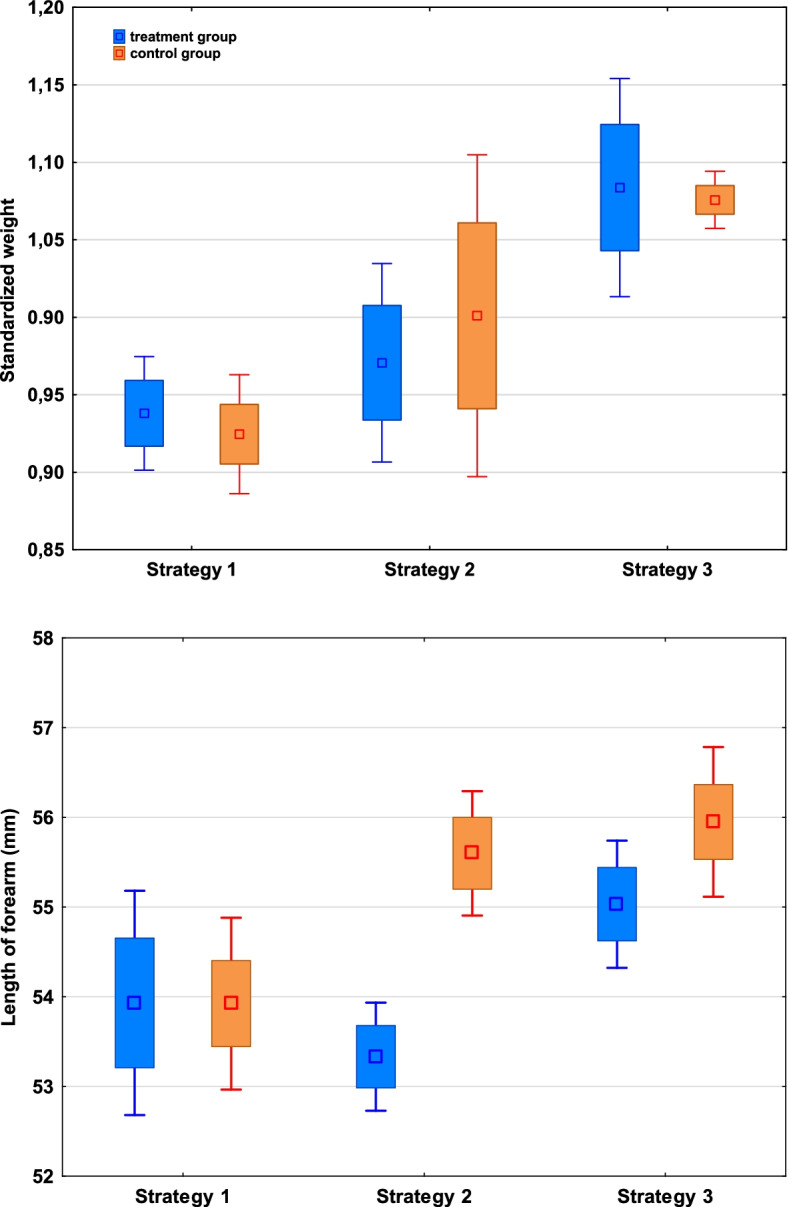
Standardised weight (**A**) and length of antebrachium (**B**) of females at the end of hibernation (14th April 2020). Blue = treatment group, orange = control group, square = mean, box = standard error, whisker = standard deviation

Standardised body weights differed only at the end of hibernation (14th April 2020) when the bats were divided into the two experimental groups, and it was these body weights, rather than hibernation length, that determined the female’s subsequent optimal reproduction strategy (Table 3), with lightest females giving birth to single young (Strategy 1), heaviest females always giving birth to twins (Strategy 3) and females of intermediate weight initially developing two foetuses with one resorbed during gestation (Strategy 2). The period of foetus resorption occurred between 27th April and 27th May 2020, when the standardised body weight of Strategy 2 females changed significantly (Table 4). While females with assumed resorption had intermediate weights that did not differ significantly from the other two groups (Strategy 1 and Strategy 3) at the first experimental measurement (27th April 2020), they differed significantly from Strategy 3 (twins) females at the next measurement (27th May 2020; LSD post-hoc test results). The significantly higher body weight of females with twins persisted up to 1 month after delivery (29th July 2020; Table 4). Within the Strategy 2 group (assumed resorption), we also observed a significant difference in antebrachium length between the control and experimental groups (t = − 4.275, *p* = 0.013), despite there being no significant difference in absolute (t = − 1.519, *p* = 0.204) or standardised (t = − 0.430, *p* = 0.689) body weight. The assumed resorption occurred in larger (longer forearm) but lighter females than Strategy 3 females (twins) from the control group, and in smaller (shorter forearm) but heavier than Strategy 1 females (single young) from the experimental group (Fig. 3).

**Table 3:** ANOVA of standardised body weight at the end of hibernation (14th April 2020) confirmed that body weight, and not hibernation length, determined female optimal reproduction strategy

Effect	SS	Degree of freedom	MS	F	***p***
Intercept	19.649	1	19.649	6334.54	**< 0.001**
Length of hibernation (Hib)	0.001	1	0.001	0.01	0.912
Reproductive strategy (Strat)	0.079	2	0.040	12.74	**0.001**
Error	0.050	16	0.003

**Table 4:** Probabilities of LSD post-hoc comparisons of body weight at three stages in the reproduction cycle. Bold numbers are significantly different at *p* < 0.05 or *p* < 0.01

Date	Strategy 1 vs. Strategy 2	Strategy 1 vs. Strategy 3	Strategy 2 vs. Strategy 3	Significant differences
April 27th	0.219	**0.008**	0.124	singleton vs. twins
May 27th	0.644	**0.016**	**0.008**	twins vs. all other strategies
June 29th	0.511	**0.022**	**0.007**	twins vs. all other strategies

**Fig. 4:**
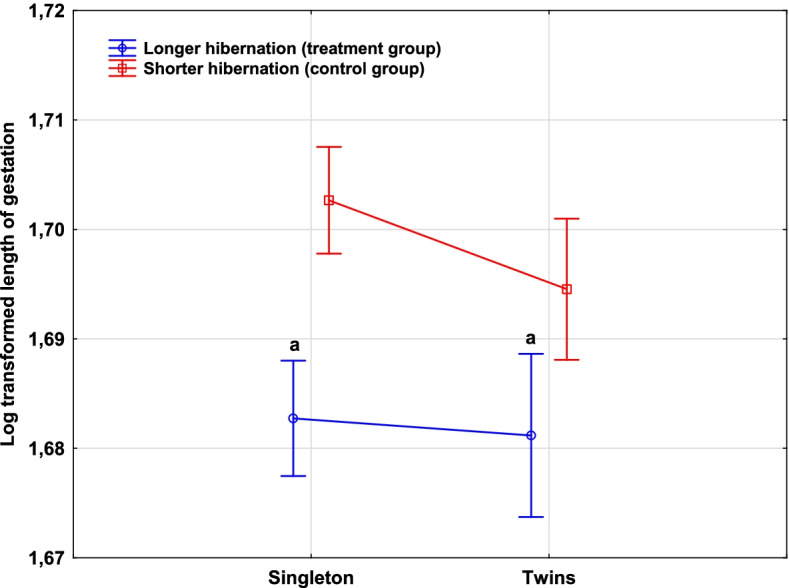
Length of gestation (log-transformed) in *Nyctalus noctula* females giving birth to one or two pups. Red = control group, blue = treatment group. Females from the treatment group shortened their gestation period by approximately 2 days. Values marked by the same letter do not differ statistically

Length of gestation differed significantly between the two experimental groups, with the longer hibernating females (treatment group) shortening their gestation period by approximately 2 days. Post-hoc comparisons indicated a significant difference between gestation length in the control group, with females with twins having a shorter gestation period than females with single young (Fig. 4).

## Discussion

Owing to the significant ecological and physiological constraints of reproduction, temperate bat species usually produce just one offspring per year [6]. However, some bats, including *N. noctula*, are capable of bearing twin offspring in a single litter [7]. As reproduction is the most expensive of life-history traits, no more than 20% of females tend to give birth to twins under natural conditions [38], though this may increase up to 88% in captivity [28]. The proportion of twins in our study was also higher (35% of females) than in nature, most likely due to the stable microclimatic conditions and stable access to food. Maximum fecundity, i.e. delivery of twins, is dependent on the condition of females in the early post-hibernation period and the maintenance of condition during pregnancy. Any increase in current reproductive output (maximum fecundity) is likely to occur only at the cost of future survivorship or future reproductive output, i.e. females are faced with the decision of investing in twins (actual reproduction success) or in single offspring (potential higher reproduction success in the future). It is likely that the second group defined on the basis of progesterone concentration pattern represents most probably females with supposed resorption of a single embryo.

A female’s outcome about actual reproduction strategy will depend primarily on her fat reserves (perceived as body weight) after the emergence from hibernation [39, 40], as this influences both the amount of energy she can invest into embryo development, and the concentration of progesterone, the hormone that allows pregnancy to be maintained. In such cases, optimal conditions for successful reproduction will be ensured by utilising a hibernation strategy that maintains maximum fat reserves (cf. the “Thrifty Female Hypothesis”), [41] and through optimal timing of emergence from hibernation. Adult *M. lucifugus* females, for example, enter hibernation with higher fat reserves and spend these reserves more slowly than males during winter, presumably so that they can emerge from hibernation in good condition and initiate pregnancy [42]. The heaviest females in our study gave birth to twins regardless of length of hibernation and maximised their actual reproductive success. At the same time, we found that birth of twins was not observed in heavier but smaller females as a consequence of their longer hibernation (Strategy 2 females in treatment group; cf. Fig. 3). Births late in the year led to reduced skeletal growth, high juvenile mortality and poor long-term survival potential [43, 44]. While female *M. lucifugus* emerge earlier than males, females in the best condition emerge first, possibly in order to exploit more variable but potentially warmer microclimates at maternity roosts and occasional warm spring nights for foraging [45]. These factors could expedite gestation and support juvenile survival [25, 46].

In our study, it is likely that the lighter females with shorter hibernation (control group) resorbed one embryo in order to reduce their actual reproduction costs. It is often the case that female mammals can terminate a reproductive attempt that has a low probability of success and threatens future reproduction. Cases of embryonal mortality in bats may occur due to poor physical condition, resulting in blocking of egg implantation, resorbing of embryos or abortion of foetuses [27]. When abortion is not associated with expulsion of the products of conception from the uterus, the ensuing pyometra may result in death of the female [47]. In the big brown bat *Eptesicus fuscus*, implantation of five embryos has been demonstrated, though none of the females studied gave birth to more than two young. In the same species, occurrence of a developing foetus in the right uterine horn and a resorbed embryo in the left horn has also been confirmed [48]. Nevertheless, termination of gestation remains somewhat debatable in wild animals as stress and other external ecological conditions, such as food deprivation, low food quality, high population density and/or genetic defects, can also play an important role [4].

Fat reserves are important as the basic building block for production of progesterone [39]. In some mammals, the amount of progesterone in the blood is known to reflect the number of embryos [49]. We found that progesterone concentration in the early phase of gestation (Stage 1) was positively correlated with post-hibernation body weight. We were also able to describe two different patterns of progesterone development during gestation, enabling us to differentiate different reproduction strategies, i.e. females pregnant with single young, twins or single young after assumed embryo resorption. On the other hand, maximum concentration of blood progesterone (between 500 and 700 ng/ml; cf. Fig. 1) did not differ between the three strategies, suggesting that this represents the physiological value necessary for successful gestation. High progesterone concentrations in tree-dwelling *N. noctula* may reflect a need of a stronger physiological protection against embryo abortion due to the high impact of changing external conditions during gestation. Progesterone concentrations in *M. lucifugus*, which use buildings as summer maternity colony roosts [50], only reached up to 200 ng/ml during the later stages of gestation [32, 33], and progesterone concentrations are even lower in the polyoestrous tropical Arafura large-footed bat *Myotis moluccarum*, Australian *C. gouldii* or South African populations of *M. schreibersii* [34–36].

Our results showed that females that spent 7 days longer in torpor (treatment group) gave birth to offspring an average of 4 days later than the control group. This suggests that they shortened the gestation period by two to three days in order to allow the juveniles to develop for longer during the optimal summer season with its rich food resources. In the greater horseshoe bat *Rhinolophus ferrumequinum*, pups born earlier are more likely to survive, increasing selection pressure to give birth to pups as early as possible [43]. This ability of female bats to manipulate the length of gestation has been known for some time [51], and a wide range of gestation lengths have now been recorded [13, 18, 28]. Indeed, our own estimation of 49-days, obtained under standard laboratory conditions with unlimited food and favourable temperatures, is similar to that calculated by [11]. The estimation of gestation length depends on two main factors, i.e. time of arousal from hibernation (as mentioned above) and subsequent environmental conditions [52]. On the other hand, it can be difficult to accurately determine time of natural ovulation in vespertilionid bats due to their use of sperm storage and delayed ovulation. In this study, we simply defined gestation length as the number of days between the end of hibernation (transfer to captivity) and the date of parturition (see [25, 53]). Unfortunately, there may be an element of bias in this estimation as we do not know the life history of the bats before they were transferred from their destroyed hibernation roost at the end of March. For example, the females may already have left the hibernaculum during warm nights and ovulation may have already occurred before the defined end of hibernation.

In bats, episodes of daily torpor under sub-optimal roosting conditions or periods of adverse weather will prolong gestation and delay parturition [25, 51, 54], though the length of gestation is species specific and relatively stable in mammals [55]. The use of daily torpor and pregnancy are usually considered to be mutually exclusive processes, however, their simultaneous occurrence is described in an increasing number of mammalian species [56]. In pregnant *P. pipistrellus*, it has been shown that the mean length of gestation is extended by induction of torpor at different stages during pregnancy, and that this extension is in good agreement with the period of torpor [51, 57]. Females will adopt such thermoregulatory behaviours during the gestation period in order to optimise juvenile development [26]. Pregnant females may also fall into shallower and shorter bouts of torpor and maintain a higher body temperature [58–60]. In our own study, where conditions were favourable (stable ambient temperature and food supply), there was no need for the females to utilise torpor and or social thermoregulation at all. As such, we assume that the estimated gestation length is standard for *N. noctula* and that females optimise their behaviour to keep the length of the gestation as close to optimal as possible.

## Conclusion

The correct timing of reproduction during the active season is vital for temperate zone animals, including bats. In fact, the conditions for bats are even more complicated due to their use of delayed ovulation and periods of torpor throughout the winter season. As long-lived animals with low natality, female bats must balance a trade-off between immediate and potential future reproduction. We found that *N. noctula* females utilise three reproductive strategies, one being embryo resorption that allows them to manipulate litter size, i.e. to give birth to single offspring or twins, and most probably maximise their inclusive fitness. We also estimated gestation length at around 49-days, and propose this as a species-specific standard that may be altered by external conditions. Finally, *N. noctula* females optimise their thermoregulatory behaviour in order to keep length of gestation as close to optimal as possible.

## Material and methods

### Animals and captivity management

A total of 22 female *N. noctula* were rescued at the end of March following the reconstruction of a hospital building (Velký Týnec; 49.5519731 N, 17.3375533E) and submitted for veterinary care, where they were kept for 2 weeks at a stable temperature of 6 °C in an artificial hibernaculum. This pre-experimental period allowed for standardisation of hibernation conditions prior to arousal at the beginning of the experiment. On 14th April 2020, each bat was weighed and aged, and their forearms measured, after which each was individually identified (marked by nail polish of different colour) before being randomly divided into two groups using a random number generator. The control group, with a shorter hibernation period, was placed in a wooden box and kept at an ambient temperature of 22 °C, while the treatment group was returned to artificial hibernation and then placed in the same wooden box a week later, i.e. on 21st April 2020, simulating a longer hibernation. Females numbered 4 and 12 died from unknown causes during captivity, leaving just nine bats in the treatment group. Active animals were fed with mealworms *Tenebrio molitor* and/or superworms *Zophobas morio* on a daily basis, combined with veterinary diet canine/feline convalescence support mix (Royal Canin, France). In addition, an immune-booster complex multivitamin paste was administered twice a week (H. von Gimborn GMBH, Germany) to supplement mineral elements. Water was freely available ad libitum.

### Blood sampling and progesterone analysis

Blood was collected from each bat on three occasions, representing 1) the initial stage of pregnancy (7 days after the end of hibernation), when ovulation and nesting of the fertilised egg occurs (April – Stage 1); 2) the late stage of pregnancy (May – Stage 2); and 3) the period shortly after delivery (June – Stage 3). Blood was drawn from uropathagial vessel as described in [61]. Briefly, the collection site was disinfected with alcohol, then about 2 μl of heparin was applied to the skin and the blood vessel punctured with a sterile needle. The blood sample was collected using an automatic pipette with a sterile heparinised tip and placed into a prepared tube. This method can provide up to 200 μl of blood, i.e. the maximum volume allowed for safe collection from a mammal the size of *N. noctula* bat [37]. After collection, the puncture site was compressed and sealed with a drop of absorbable surgical tissue glue (Surgibond, SMI AG, Belgium) and the animal was given food supplemented with glucose. The plasma was separated through centrifugation and stored in a freezer at − 20 °C until further analysis. Progesterone concentrations were determined using a Progesterone ELISA kit (Enzo Life Sciences Inc., USA), following the manufacturer’s instructions.

### Statistical analysis

Normal distribution of variables was tested using the Shapiro-Wilk test. All variables were normally distributed with the exception of gestation length, which was subsequently log transformed. Gestation length was calculated as the number of days between the end of hibernation and date of birth. The t-test and ANOVA were used for comparing body size (forearm length and weight) between the two age and treatment groups. While the control and treatment groups did not differ in forearm length, they differed in body weight at the time of division into experimental groups (14th April 2020); thus, the body weight of each group was standardised by dividing it by the particular subsample mean. We defined the clusters of females under study with the greatest possible distinction using k-means clustering on progesterone data, with three clusters expected for calculation based on different progesterone patterns, i.e. single and/or twins and embryo resorption. Differences in progesterone concentration and standardised body weight between the three types of above-defined groups and length of hibernation were compared using Main Effects ANOVA, with post-hoc comparisons using the Fisher LSD test. Similarly, gestation length between the females with one or two pups was compared using Main Effects ANOVA evaluating the impact of treatment and the number of juveniles born (single vs. twins). The relationship between body weight and progesterone concentration was assessed using the Pearson’s correlation coefficient. For analysis was used software Statistica for Windows®13.5 (StatSoft, Inc., Tulsa, OK, USA).

## Supplementary Information

**Additional file 1: Supplementary Table S1.** Data on body weight, progesterone concentration and gestation length of *Nyctalus noctula.*

## Data Availability

The datasets used and/or analysed during the current study are available from the corresponding author on reasonable request. Log-transformed data on gestation length, standardized body weight and progesterone concentrations accompany this published article as additional file Table S1.
