# Supplementary material for: One or two pups - optimal reproduction strategies of common noctule females
Source: BMC Zool. 2022 Apr 2;7:18. doi: 10.1186/s40850-022-00119-8 (PMC10127298; doi:10.1186/s40850-022-00119-8)
Supplement: Supplementary file 1 — Additional file 1: Supplementary Table S1. Data on body weight, progesterone concentration and gestation length of Nyctalus noctula. [file 40850_2022_119_MOESM1_ESM.docx]

**One or two pups - optimal reproduction strategies of Common Noctule females**

**Supplementary Table S1**

Data on body weight, progesterone concentration and gestation length of *Nyctalus noctula*

| **ID** | **Lenght of hibernation** | **Standardized body weight** | **BMI** | **Number of pups** | **Progesterone concentration - Stage 1** | **Progesterone concentration - Stage 2** | **Progesterone concentration - Stage 3** | **Log-transformed gestation length** |
| --- | --- | --- | --- | --- | --- | --- | --- | --- |
| NN1 | b | 0.946 | 0.361 | 1 | 174.9088 | 605.6349 | 76.31794 | 1.690 |
| NN2 | a | 0.941 | 0.375 | 1 | 360.84 | 372.7098 | 47.92604 | 1.699 |
| NN3 | b | 0.898 | 0.351 | 1 | 461.8403 | 717.8024 | 81.09268 | 1.681 |
| NN5 | b | 1.043 | 0.395 | 2 | 529.9439 | 750.4675 | 490.7348 | 1.690 |
| NN6 | b | 1.019 | 0.393 | 1 | 438.1783 | 383.4156 | 138.3266 | 1.681 |
| NN7 | a | 0.964 | 0.405 | 1 | 388.1 | 697.7597 | 111.631 | 1.708 |
| NN8 | b | 0.898 | 0.351 | 1 | 628.0929 | 765.8027 | 94.95212 | 1.681 |
| NN9 | b | 0.97 | 0.362 | 1 | 327.4514 | 588.7242 | 72.40785 | 1.681 |
| NN10 | a | 0.941 | 0.383 | 1 | 478.97 | 429.4038 | 190.4183 | 1.699 |
| NN11 | a | 1.076 | 0.431 | 2 | 581.62 | 640.9275 | 388.0975 | 1.681 |
| NN13 | b | 0.995 | 0.380 | 1 | 521.437 | 283.0706 | 235.9553 | 1.681 |
| NN14 | b | 1.043 | 0.391 | 2 | 703.4284 | 475.7445 | 303.6314 | 1.672 |
| NN15 | a | 0.941 | 0.383 | 1 | 332.79 | 738.4208 | 276.2821 | 1.699 |
| NN16 | a | 1.076 | 0.419 | 2 | 567.67 | 397.6334 | 267.4834 | 1.699 |
| NN17 | a | 0.874 | 0.366 | 1 | 303.22 | 613.0302 | 120.5501 | 1.708 |
| NN18 | a | 0.919 | 0.374 | 1 | 131.24 | 402.4888 | 197.4794 | 1.699 |
| NN19 | a | 1.121 | 0.446 | 1 | 670.09 | 498.7408 | 99.27312 | 1.708 |
| NN20 | b | 1.165 | 0.430 | 2 | 623.0313 | 422.5108 | 87.57188 | 1.681 |
| NN21 | a | 1.098 | 0.441 | 2 | 534.25 | 469.3749 | 315.7431 | 1.699 |
| NN22 | a | 1.053 | 0.423 | 2 | 651.38 | 683.7871 | 191.9653 | 1.699 |
